# Central Venous Pressure (CVP) Reduction Associated With Higher Cardiac Output (CO) Favors Good Prognosis of Circulatory Shock: A Single-Center, Retrospective Cohort Study

**DOI:** 10.3389/fmed.2019.00216

**Published:** 2019-10-15

**Authors:** Longxiang Su, Pan Pan, Dongkai Li, Qing Zhang, Xiang Zhou, Yun Long, Xiaoting Wang, Dawei Liu

**Affiliations:** ^1^Department of Critical Care Medicine, Peking Union Medical College Hospital, Peking Union Medical College, Chinese Academy of Medical Sciences, Beijing, China; ^2^Department of Critical Care Medicine, Chinese PLA General Hospital, Beijing, China

**Keywords:** cardiac output (CO), central venous pressure (CVP), hemodynamics, prognosis, circulatory shock, renal function

## Abstract

**Background:** The Frank-Starling curve is the basis of hemodynamics. Changes in cardiac output (CO) caused by central venous pressure (CVP) are the most important concerns in the treatment of critically ill patients.

**Objectives:** To explore the use of CVP and its relevant mechanisms with respect to CO in the clinic.

**Methods:** A total of 134 patients with circulatory shock were retrospectively included and analyzed. Hemodynamic data were recorded and analyzed at PICCO initiation and 24 h after PICCO. Data regarding 28-day mortality and renal function were also collected.

**Results:** The patients were divided into a CVP↑+ CO↑ group (*n* = 23), a CVP↑+ CO↓ group (*n* = 29), a CVP↓+ CO↑ group (*n* = 44), and a CVP↓+ CO↓ group (*n* = 38) based on values at PICCO initiation and 24 h after PICCO. *Post- hoc* tests showed that the CVP↓+ CO↑ group had a higher 28-day survival than the other groups [log-rank (Mantel-Cox) = 8.758, 95%, CI, 20.112–23.499, *P* = 0.033]. In terms of hemodynamic characteristics, the CVP↓+ CO↑ group had a lower cardiac function index (CFI) (4.1 ± 1.4/min) and higher extravascular lung water index (EVLWI) (11.0 ± 4.7 ml/kg) at PICCO initiation. This group used more cardiotonic drugs (77.3%, *P* < 0.001) and had a negative fluid balance (−780.4 ± 1720.6 ml/24 h, *P* = 0.018) 24 h after PICCO than the other three groups. Cardiotonic drug use and dehydration treatment were associated with increased CFI (from 4.1 ± 1.4 /min to 4.5 ± 1.3/min, *P* = 0.07) and reduced ELVWI (from 11.0 ± 4.7 ml/kg to 9.0 ± 3.5 ml/kg, *P* = 0.029). Renal function tests showed that SCr and BUN levels in the CVP↓+ CO↑ group were significantly improved (SCr from 197.1 ± 128.9 mmol/L to 154.4 ± 90.8 mmol/L; BUN from 14.3 μmol/L ± 7.3 to 11.6 ± 7.0 μmol/L, *P* < 0.05).

**Conclusions:** Lower CVP was associated with increased CO, which may improve the 28-day prognosis in patients with circulatory shock. Notably, higher CO derived from lower CVP may also contribute to renal function improvement.

## Introduction

Central venous pressure (CVP) is the pressure in the thoracic vena cava near the right atrium. CVP is an important factor in critical care medicine because it can be used to estimate a patient's fluid volume status, assess cardiac function, and gauge how well the right ventricle of the heart is functioning (1). Due to the emphasis on early fluid resuscitation, excessive fluid resuscitation is more common in clinical practice (2). As a preload parameter for assessing volume capacity, CVP may be abnormally elevated due to acute right or left heart failure and excessive increases in external pressure (including pericardial pressure, intrathoracic pressure, and abdominal pressure) (3). Recent studies have challenged the value of monitoring elevated CVP in critically ill patients, including those with cardiovascular dysfunction, renal failure, or acute respiratory distress syndrome (ARDS) (4–6). Some studies have concluded that elevated CVP is associated with increased mortality in critically ill patients (7, 8). A review of previous studies confirmed that elevated CVP indicates poor outcomes (9). The main mechanisms underlying the harm caused by elevated CVP may include impeded venous return as well as accompanying lung edema and splanchnic congestion that may further worsen potential organ failure (10). Conversely, early reductions in CVP during treatment may help maintain good organ function and result in a higher survival rate (11). CVP has been reported to be very low under normal physiological conditions (12). Therefore, perhaps CVP should remain as low as possible in critically ill patients.

Based on the Frank-Starling mechanism and venous return (VR) theory proposed by Guyton, VR should match cardiac output (CO) as determined by the mean systemic filling pressure (MSFP) and the CVP gradient (10, 13). Changes in CO due to CVP are important concerns for the treatment of critically ill patients. Notably, increases in CO corresponding with increases in CVP are indicative of responses to fluid challenge. However, decreases in CO with increases in CVP are indicative of a primary decrease in cardiac function, whereas reductions in CO with decreases in CVP are indicative of a primary decrease in return function and likely, a decrease in volume. Increases in CO with decreases in CVP are indicative of improvement of heart function and pulmonary circulation, especially right heart function. Nevertheless, increases in CO with decreases in CVP occur in routine clinical work, and we speculate that these patients may benefit from this phenomenon. In this study, we selected patients with circulatory shock to demonstrate the influence of the relationship between CVP and CO on survival and to explore how and why this hemodynamic scenario has a beneficial effect.

## Materials and Methods

This study is retrospective, and all the patients authorized us to use their clinical data. The research protocol was reviewed and approved by the Ethics Committee of Peking Union Medical College Hospital (PUMCH-S616). When PICCO catherization was needed, the patients or their family members were fully informed of the details, and they signed informed consent forms.

### Participant Inclusion

**Figure 1:**
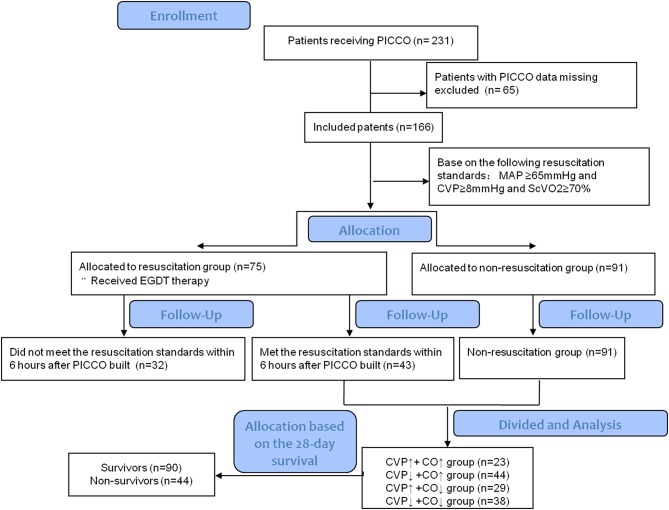
The flowchart of the PICCO patients included in this study.

The Critical Care Monitor System and Administrative Database of Peking Union Medical College Hospital was built in 2013 (14). This database integrates basic patient data, clinical monitoring and laboratory data, treatment information, nursing information, and many other factors. We retrospectively collected all data from patients with circulatory shock as follows (15): (1) sustained hypotension, systolic arterial pressure <90 mm Hg or mean arterial pressure <65 mm Hg; (2) clinical signs of tissue hypoperfusion (abnormal cutaneous, renal, or neurologic perfusion); and (3) hyperlactatemia (blood lactate ≥2 mmol/L). We also recorded whether PICCO catheterization and monitoring were performed during treatment. Ultimately, 231 patients with circulatory shock who underwent PICCO and were treated in the Department of Critical Care Medicine, Peking Union Medical College Hospital, from August 2013 to December 2015 were included and analyzed. The relevant data from PICCO initiation, 6 h after PICCO and 24 h after PICCO were automatically collected from the system and database for analysis, and the relevant clinical parameters were also recorded. The flow chart of this study is shown in Figure 1.

### Clinical Treatment Programs

The resuscitation standards used here were designed to maintain a mean arterial pressure (MAP) ≥ 65 mmHg, CVP 8–12 mmHg and ScVO_2_ ≥ 70% (SvO_2_ ≥ 65%) based on early goal-directed therapy (EGDT) (16).

First, we worked hard to ensure that patients achieved EGDT targets within 6 h after PICCO initiation. The specific measures were as follows: (1) assessed volume responsiveness and continuous fluid resuscitation to achieve and maintain CVP 8–12 mmHg; (2) if the negative fluid challenge or the blood pressure did not increase after the volume responsiveness, vasoconstrictor drugs were used to achieve a MAP ≥65 mmHg; (3) if ScvO_2_ < 70% (SvO_2_ < 65%) and hematocrit <30%, blood transfusion was adopted; and (4) if ScvO_2_ < 70% (SvO_2_ < 65%) and hematocrit ≥30%, inotropic drugs were used to achieve ScvO_2_ ≥ 70% (SvO_2_ ≥ 65%). After the resuscitation was completed, a restrictive fluid management strategy was used; if satisfactory perfusion indicators were obtained (e.g., MAP ≥ 65 mmHg, CVP 8–12 mmHg, ScVO_2_ ≥ 70% (SvO_2_ ≥ 65%), P_(v−a)_CO_2_ < 6, and decreased lactate levels), limited fluid therapy was used during treatment. The strategy to control fluid balance measures did not rule out the use of artificial means of dehydration, including diuretics, and renal replacement therapy.

### Hemodynamic Monitoring Methods

1. CVP measurement: Using an indwelling central venous catheter via the internal jugular or subclavian vein, a pressure sensor was connected to a monitor (Philips). While the patients were supine, the sensor was positioned in the axillary line at the fourth intercostal level. The pressure waveform and CVP values were read at the end expiratory time and recorded by ICU physicians.
2. CO, cardiac output index (CI), cardiac function index (CFI), extravascular lung water index (EVLWI), and other hemodynamic data were measured by the thermodilution method. The femoral PICCO™ catheter was connected to a module PICCO plus® system (Pulsion Medical Systems, Munich, Germany) and recalibrated for the detection of hemodynamic data in accordance with the manufacturer's instructions. To ensure the accuracy of the measurements, transpulmonary thermodilution measurements were acquired in triplicate by 15 ml 0–4°C normal iced saline solution bolus injection through a central vein to obtain an average value, which was used for statistical analysis.
3. P(v-a)CO_2_, ScvO_2_ and lactate: Arterial blood and superior vena cava blood were collected simultaneously. Arterial and venous blood samples were tested by a blood gas analyzer (GEM® PREMIER™ 3000). ScvO_2_ and lactate were read directly from the results. P_(v−a)_CO_2_ was calculated by PvCO_2_-PaCO_2_.

### Data Collection

Basic clinical characteristics were collected, including underlying disease, source of infection, organ function, APACHE II and SOFA scores, mechanical ventilation application, vasoactive drug use, fluid balance, hemodynamic parameters, tissue perfusion index, pulmonary vascular permeability index, and 28-day mortality. All the data came from the Critical Care Monitor System and Administrative Database of Peking Union Medical College Hospital (14).

### Statistical Analysis

The data distribution test and the homogeneity of variance test were performed on the data (17). The results for continuous variables with normal distributions are given as the means ± standard deviations (SD). Student's *t*-test and analysis of variance (ANOVA) were used to compare means between two groups or three or more groups, respectively. The results for qualitative variables were expressed as percentages and compared between groups using a chi-square test. Survival curves up to day 28 were estimated using the Kaplan-Meier method, and the log-rank (Mantel-Cox) test was used to estimate differences between the groups. Repeated measures ANOVA was used to describe the dynamic changes in renal function among different groups at different time points after PICCO catheterization (PICCO initial, 24, 48, and 72 h after PICCO). Statistical analyses were performed with SPSS version 13.0 (SPSS Inc., Chicago, IL, USA).

## Results

### Patient Baseline Characteristics

In this study, we included 231 patients with circulatory shock who underwent PICCO during the observation period. Of these patients, 65 were excluded due to any missing data at 24 h after PICCO. Complete data were available for 166 patients. On the basis of the hemodynamic characteristics from PICCO, circulatory shock was classified in 166 patients as follows: hypovolemia (8, 4.8%), cardiogenic factors (65, 39.16%), obstruction (21, 12.65%), or distributive factors (72, 43.37%). These patients were divided into a resuscitation group (*n* = 75) that required resuscitation treatment and a non-resuscitation group (*n* = 91) that did not need resuscitation treatment based on the EGDT criterion. A total of 43 patients in the resuscitation group achieved MAP ≥ 65 mmHg, CVP ≥ 8 mmHg, and ScVO_2_ ≥ 70% within the initial 6 h resuscitation treatment based on the 6 h “sepsis bundle.” The remaining 32 patients did not meet the resuscitation standard and were excluded. Therefore, 91 patients from the non-resuscitation group and 43 patients who met the resuscitation standards within the initial 6 h period were included in the subsequent analysis. Based on the changes and the relationship between CO and CVP at the start of PICCO and 24 h after PICCO, these 134 patients were further divided into four groups according to the differences in CVP and CO between PICCO initiation and 24 h after PICCO: a CVP↑+ CO↑ group, a CVP↑+ CO↓ group, a CVP↓+ CO↑ group, and a CVP↓+ CO↓ group (Figure 1). The demographic and clinical characteristics of the included patients at the start of PICCO are summarized in Table 1. The CVP↑+CO↑ group had significantly higher SOFA scores than the CVP↓+CO↓ group (*P* < 0.05). The CVP↓+CO↑ group had the lowest 28-day mortality rate (*P* < 0.05). In addition, there were no significant differences in terms of age, sex, sources of infection (excluding pulmonary), pathogens detected, frequency of continuous renal replacement therapy (CRRT) and mechanical ventilation (MV), and respiratory function indices, including PEEP, FiO_2_%, and PO_2_/FiO_2_%, among the five groups.

**Table 1:** The general characteristics of patients involved in this study at the initiation of PICCO.

**Characteristics**	**Excluded group**	**CVP↑+ CO↑ group**	**CVP↓+ CO↑ group**	**CVP↑+ CO↓ group**	**CVP↓+ CO↓ group**	***P*-value**
	***n* = 32**	***n* = 23**	***n* = 44**	***n* = 29**	***n* = 38**	
Age (years)	57.6 ± 19.6	55.0 ± 16.8	50.9 ± 14.0	56.8 ± 18.6	60.2 ± 15.9	0.278
**Sex**, ***n*** **(%)**						
Male	16 (50)	12 (52.2)	20 (45.5)	15 (51.7)	19 (50)	0.981
Female	16 (50)	11 (47.8)	24 (54.5)	14 (48.3)	19 (50)	
Circulatory shock						*P* < 0.001
Hypovolemia	5 (15.6)	2 (8.7)	0 (0)	0 (0)	1 (2.6)	
Cardiogenic factors	11 (34.4)	2 (8.7)	33 (75)	11 (37.9)	8 (21.1)	
Obstruction	2 (6.2)	2 (8.7)	0 (0)	16 (55.2)	1 (2.6)	
Distributive factors	14 (43.8)	17 (73.9)	11 (25)	2 (6.9)	28 (73.7)	
APACHE II score	26.7 ± 8.1	30.2 ± 7.8	26.7 ± 10.6	25.7 ± 8.6	27.0 ± 8.4	0.383
SOFA score	13.3 ± 3.2	14.0 ± 3.3	13.8 ± 3.9	12.0 ± 4.1	11.7 ± 4.3	0.044
Mortality, *n* (%)	16 (50)	9 (39.1)	7 (15.9)	11 (37.9)	17 (44.7)	0.019
FiO_2_%	44.5 ± 20.3	50.0 ± 14.7	47.3 ± 16.6	47.0 ± 13.4	19.0 ± 9.1	0.799
PaO2/FiO_2_	211.0 (164.4–38.07)	241.5 (169.4–295.6)	224.1 (151.6–340.4)	274.3 (152.2–343.6)	219.0 (151.6–340.4)	0.88
MV, *n* (%)	30 (93.8)	22 (95.7)	39 (88.6)	29 (100)	35 (94.6)	0.381
PEEP (H_2_O)	7.9 ± 5.0	8.9 ± 2.9	6.7 ± 2.3	8.1 ± 4.1	7.9 ± 3.2	0.862
CRRT, *n* (%)	16 (50)	11 (47.8)	24 (54.5)	15 (51.7)	14 (40.0)	0.775
**Underlying disease**, ***n*** **(%)**						
Hypertension	10 (31.25)	9 (39.1)	15 (34.1)	12 (41.4)	12 (31.6)	*P* > 0.05
Diabetes mellitus	9 (28.1)	7 (30.4)	10 (22.7)	10 (34.5)	13 (34.2)	*P* > 0.05
Chronic cardiac dysfunction^a^	11 (34.4)	7 (30.4)	16 (36.3)	11 (37.9)	12 (31.6)	*P* > 0.05
Obstructive ventilatory impairment^b^	10 (31.3)	6 (26.1)	12 (27.2)	9 (31)	8 (21.1)	*P* > 0.05
Chronic renal insufficiency^c^	8 (25)	7 (30.4)	11 (25)	7 (26.9)	9 (23.7)	*P* > 0.05
Chronic hepatic insufficiency^d^	5 (15.6)	3 (13)	7 (15.9)	5 (17.2)	5 (13.2)	*P* > 0.05
Nervous system disease^e^	7 (21.9)	5 (21.7)	10 (22.7)	7 (24.1)	7 (18.4)	*P* > 0.05
Immunosuppressed condition	3 (9.3)	1 (4.3)	2 (4.5)	0 (0)	1 (2.6)	*P* > 0.05

*Quantitative data are expressed as the mean ± SD or median interquartile (25–75)%. Qualitative data are expressed as n (%)*.

*APACHE II, Acute Physiology and Chronic Health Evaluation II; SOFA, sequential organ failure assessment; PaO_2_, arterial oxygen pressure; FiO_2_, fraction of inspiration oxygen; MV, mechanical ventilation; PEEP, positive end expiratory pressure; CRRT, continuous renal replacement therapy. PaO2, arterial oxygen pressure; FiO_2_, fraction of inspiration oxygen; MV, mechanical ventilation; PEEP, positive end expiratory pressure*.

a *All patients corresponded to the New York Heart Association (NYHA) standards of level II or higher*.

b *In an obstructive type of ventilatory impairment, the impairment is the result of an airway obstruction due to bronchial obstruction, as in asthma, or obstruction in other parts of the airway, e.g., laryngeal edema or carcinoma. This type of ventilatory impairment is characterized by a reduction in VC and FEV1. The relative reduction in FEV1 is greater than the reduction in VC, and hence, there is a reduction in the forced expiratory ratio, FEV1/FVC, which decreases the value below 0.70*.

c *All patients were receiving long-term hemodialysis*.

d *As described according to APACHE II criteria, biopsy-proven cirrhosis and documented portal hypertension, episodes of past upper gastrointestinal bleeding attributed to portal hypertension, or prior episodes of hepatic failure/encephalopathy/coma*.

e *The condition may be an inherited metabolic disorder; the result of damage from an infection, a degenerative condition, stroke, a brain tumor or other problem; or the product of unknown or multiple factors*.

### 24 h CVP Dynamic Changes and 28-day Mortality

**Figure 2:**
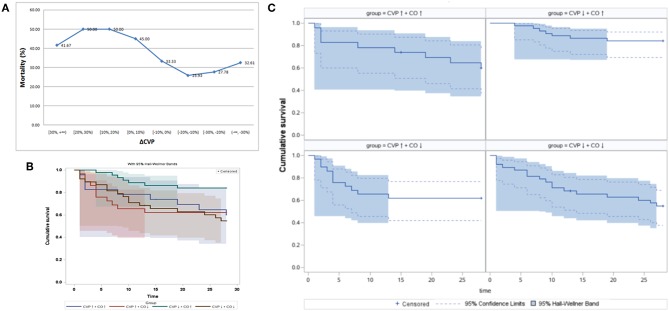
The 24 h CVP dynamic changes and 28-day mortality. **(A)** The 28-day mortality rate showed a downward trend with the reduction in CVP after 24 h PICCO. Panels **(B,C)** show shows the Kaplan-Meier analyses of 28-day survival probabilities when resuscitation standards were met. Survival was measured among the CVP↑ + CO↑ group, CVP↑ + CO↓ group, CVP↓ + CO↑ group, and CVP↓ + CO↓ group based on the changes in CO and CVP at the initiation and 24 h after PICCO.

Based on the 24 h CVP dynamic changes, all patients (*n* = 166) were divided into eight stratifications by every 10% increase and decrease in CVP. The 28-day mortality rates for these eight stratifications are shown in Figure 2A. Higher CVP was associated with a poor outcome, and lower CVP indicated a lower mortality rate (Figure 2A). The CVP↓+CO↑ group had the lowest mortality rate (Table 1). Therefore, the effect of reduced CVP on survival was further analyzed. *Post-hoc* tests showed that there were statistically significant differences in 28-day survival rates among the CVP↑+ CO↑, CVP↑+ CO↓, CVP↓+ CO↑, and CVP↓+ CO↓ groups [log-rank (Mantel-Cox) = 8.758, 95%, CI, 20.112–23.499, *P* = 0.033] (Figures 2B,C). The CVP↓+ CO↑ group had a higher 28-day survival rate than the other three groups.

### Hemodynamic Characteristics of the Patient Benefits From CVP Decreases

**Figure 3:**
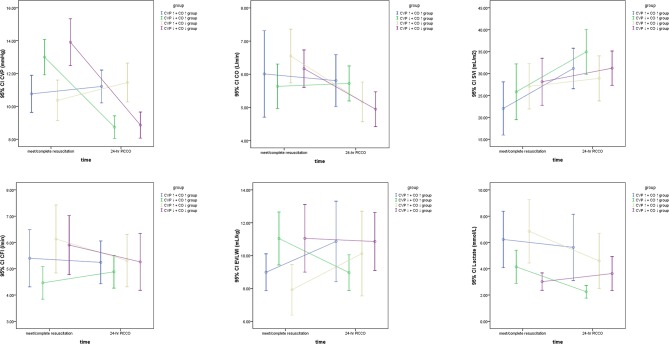
The significant hemodynamic parameters of the CVP↑+ CO↑ group, CVP↑+ CO↓ group, CVP↓+ CO↑ group, and CVP↓+ CO↓ group based on the dynamic changes in CO and CVP at the initiation and 24 h after PICCO.

The hemodynamic characteristics of all included patients when they met the resuscitation standards or completed resuscitation are shown in Table 2. Six hours after resuscitation therapy, the excluded group did not achieve satisfactory parametric levels, e.g., ScvO_2_ was significantly lower than that in the other groups (*P* < 0.05). Simultaneously, the excluded group had higher P_(v−a)_CO_2_, systemic vascular resistance index (SVRI), and EVLWI and lower CO, CI, stroke volume index (SVI), global ejection fraction (GEF), and CFI. Therefore, the excluded groups were excluded from the following study because they needed more time to reach a satisfactory perfusion index. According to Table 2, seven parameters, including CVP, CO, CI, GEF, CFI, EVLWI, and lactate, showed statistical significance among the CVP↑+ CO↑, CVP↑+ CO↓, CVP↓+ CO↑, and CVP↓+ CO↓ groups at PICCO initiation (*P* < 0.05). The CVP↓+ CO↑ (13.0 ± 3.1 mmHg) and CVP↓+ CO↓ (13.9 ± 4.1 mmHg) groups had higher CVP than the CVP↑+ CO↑ (10.8 ± 2.2 mmHg) and CVP↑+ CO↓ (10.4 ± 2.9 mmHg) groups (*P* < 0.05) at PICCO initiation. Twenty-four hours after PICCO, the CVP of the CVP↓+ CO↑ (8.7 ± 2.3 mmHg) and CVP↓+ CO↓ (8.9 ± 2.4 mmHg) groups decreased significantly (*P* < 0.05). Accordingly, the CO of the CVP↓+ CO↑ group increased from 4.9 ± 1.6 L/min to 5.7 ± 1.3 L/min (*P* < 0.05), while the CO of the CVP↓+ CO↓ group decreased from 6.2 ± 1.6 L/min to 4.9 ± 1.6 L/min (*P* < 0.05). The CI showed the same trend as the CO. The relationship between CVP and CO changes was accompanied by changes in CFI, EVLWI, and lactate (Figure 3). The CVP↓+ CO↑ group had lower CFI than the other three groups when they met the resuscitation standards or completed resuscitation (CVP↑+ CO↑ group vs. CVP↑+ CO↓ group vs. CVP↓+ CO↑ group vs. CVP↓+ CO↓ group: 5.0 ± 1.9/min vs. 4.8 ± 1.2/min vs. 4.1 ± 1.4/min vs. 4.8 ± 1.6/min, *P* < 0.05). The CFIs of the CVP↑+ CO↑, CVP↑+ CO↓, CVP↓+ CO↑, and CVP↓+ CO↓ groups were 5.2 ± 1.9/min, 4.7 ± 1.2/min, 4.5 ± 1.3/min, and 4.4 ± 1.5/min 24 h after PICCO, respectively. The CFI increase in the CVP↓+ CO↑ group at initiation and 24 h after PICCO showed a trend that was not significant (*P* = 0.07). In addition, the CVP↓+ CO↑ and CVP↓+ CO↓ groups had higher EVLWI than the other groups (*P* < 0.05). However, only the CVP↓+ CO↑ group had lower EVLWI at 24 h after PICCO compared with its EVLWI values when resuscitation standards were met or resuscitation was completed (9.0 ± 3.5 ml/kg vs. 11.0 ± 4.7 ml/kg, *P* = 0.029). The CVP↓+ CO↑ and CVP↓+ CO↓ groups had lower lactate levels than the other two groups (*P* < 0.05). With the exception of the CVP↓+ CO↓ group, the other three groups showed a decreasing trend in lactate levels. The decreases in lactate levels in the CVP↓+ CO↑ [1.8 (1.1–2.8) mmol/L] and CVP↑+ CO↓ [2.4 (1.2–7.2) mmol/L] groups 24 h after PICCO were statistically significant (*P* < 0.05). Regarding the pulmonary vascular permeability index (PVPI), the data from the four groups were not significantly different.

**Table 2:** The hemodynamic characteristics of all the included patients at PICCO initiation.

**Characteristics**	**Excluded group**	**CVP↑+ CO↑group**	**CVP↑+ CO↓group**	**CVP↓+ CO↑group**	**CVP↓+ CO↓group**	***P*-value**
	***n* = 32**	***n* = 23**	***n* = 29**	***n* = 44**	***n* = 38**	
**Hemodynamic variables**						
Heart rate (bpm)	108.4 ± 21.4	107.3 ± 18.3	112.5 ± 17.6	104.1 ± 20.6	105.1 ± 20.8	0.463
Mean arterial pressure (mmHg)	85.7 ± 9.6	86.7 ± 11.6	90.1 ± 10.7	90.1 ± 13.5	86.8 ± 12.3	0.391
CVP (mmHg)	11.1 ± 4.3	10.8 ± 2.2	10.4 ± 2.9	13.0 ± 3.1	14.0 ± 4.1	<0.001
CO (L/min)	4.2 ± 1.6	4.5 ± 1.6	6.1 ± 1.9	4.9 ± 1.6	6.2 ± 1.6	<0.001
CI (L/min/m^2^)	2.4 ± 0.8	2.7 ± 0.9	3.5 ± 1.0	3.0 ± 0.9	3.5 ± 0.8	0.001
SVI (mL/m^2^)	19.9 ± 7.1	26.0 ± 8.6	30.1 ± 9.7	31.2 ± 16.6	35.0 ± 10.1	0.009
GEF (%)	15.4 ± 4.8	19.1 ± 6.2	18.7 ± 5.4	16.4 ± 6.1	20.0 ± 6.7	0.014
CFI (/min)	3.9 ± 1.3	5.0 ± 1.9	4.8 ± 1.2	4.1 ± 1.4	4.8 ± 1.6	0.016
EVLWI (mL/kg)	11.7 ± 6.8	9.0 ± 2.2	7.9 ± 3.6	11.0 ± 4.7	11.1 ± 5.8	0.048
PVPI	2.3 ± 1.2	2.0 ± 0.8	1.8 ± 0.9	2.0 ± 0.9	2.2 ± 1.1	0.505
GEDVI (mL/m^2^)	675.0 ± 179.9	655.3 ± 169.4	696.3 ± 165.2	738.5 ± 155.9	745.7 ± 147.4	0.218
SVRI (dyn.sec.cm^−5^.m2)	2395.3 (1767.5–3317.1)	1992.2 (1643.1–2827.4)	1772.5 (1,405–2,535)	2,183 (1492.5–2928.0)	1677.2(1370.0–1926.3)	0.006
**Perfusion indexes**						
GAP	7.7 ± 4.8	5.6 ± 3.7	5.9 ± 2.6	5.9 ± 3.9	4.1 ± 2.2	0.005
ScvO_2_ (%)	58.7 ± 7.5	77.1 ± 5.4	76.3 ± 5.9	78.2 ± 6.2	77.6 ± 5.8	<0.001
Lactate (mmol/L)	3.4 (2.1–7.9)	5.6 (2.4–8.2)	4.9 (2.7–9.6)	2.5 (1.4–5.7)	2.5 (1.5–3.9)	<0.001
**Interventions**						
Vasoactive drugs (*n*, %)	–	23 (100)	28 (96.6)	41 (93.2)	35 (92.1)	0.527
Cardiotonic drugs (*n*, %)	–	10 (43.5)	19 (65.5)	34 (77.3)	11 (28.9)	<0.001
Total fluid (ml/24 h)	–	1329.3 ± 2600.4	531.0 ± 3974.0	−780.4 ± 1720.6	−1797.0 ± 3632.7	0.018

*Quantitative data are expressed as the mean ± SD or median interquartile (25–75)%*.

*CVP, central venous pressure; CO, cardiac output; CI, cardiac output index; SVI, stroke volume index; GEF, global ejection fraction; CFI, cardiac function index; GEDVI, global end-diastolic volume index; EVLWI, extravascular lung water index; SVRI, systemic vascular resistance index; ScvO_2_, central venous blood oxygen saturation. All the data were collected at the initiation of PICCO*.

### Intervention Process Comparisons

After the initial resuscitation was completed, the main interventions for all included patients were target blood pressure improvement, cardiac function enhancement, and fluid therapy. Vasoactive drug usage, cardiotonic drug usage, and total fluid volume within 24 h of PICCO were retrospectively collected. Comparisons were made among the CVP↑+ CO↑, CVP↑+ CO↓, CVP↓+ CO↑, and CVP↓+ CO↓ groups during the therapy process. Table 2 shows that cardiotonic drug use was quite different among these four groups. The cardiotonic drug use proportion in the CVP↓+ CO↓ group was lower than that in the CVP↑+ CO↓ and CVP↓+ CO↑ groups (*P* < 0.05). The CVP↑+ CO↑ group consumed more fluid during resuscitation, while more fluid was removed from the CVP↓+ CO↓ group (*P* < 0.05). Although the CVP↓+ CO↑ group also had a positive fluid balance, CO showed an increasing trend. The CVP↓+ CO↑ group showed an increase in CO as a result of CVP reduction because of dehydration.

### Dynamic Changes in Renal Function

**Figure 4:**
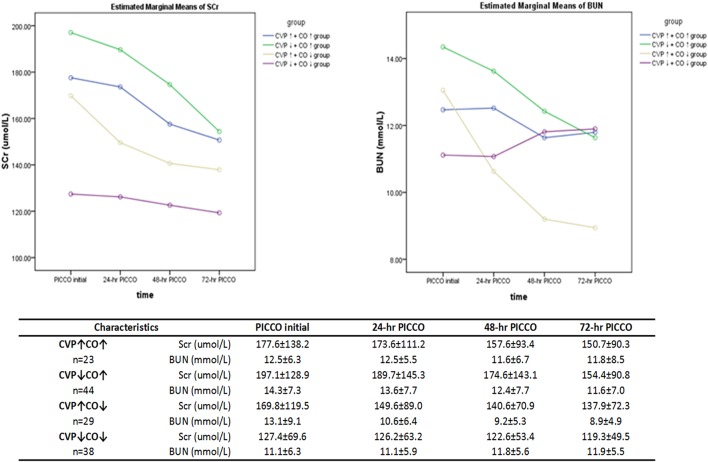
Dynamic changes in the renal function parameters (SCr and BUN) over 3 days of observation. Repeated measures analysis of variance (ANOVA) was used.

A polynomial test was used to analyze the trend curves of the dynamic changes in renal function at PICCO initiation and at 24, 48, and 72 h after PICCO catheterization (Figure 4). The curves showed that the SCr of the four groups had a tendency to decline. Three groups, excluding the CVP↓+ CO↓ group, showed declines in BUN levels. However, the SCr and BUN levels were not significantly different among the four groups during the observation period (*F* = 1.184, *P* = 0.322; F = 0.629, *P* = 0.599, respectively). There were significant differences in the SCr levels of the CVP↓+ CO↑ group and the BUN levels of the CVP↓+ CO↑ and CVP↑+CO↓ groups at various time points (*F* = 9.107, *P* = 0.03; *F* = 4.128, *P* = 0.046, respectively). SCr and BUN levels significantly improved in the CVP↓+ CO↑ group only. In addition, there was no interaction effect between the dynamics of the SCr and BUN levels in the four groups (*F* = 0.653, *P* = 0.675; *F* = 1.639, *P* = 0.169, respectively).

## Discussion

This single-center retrospective cohort study explored the relationship between CVP and prognosis. Furthermore, we explored the mechanisms of CVP changes in hemodynamics. Our results confirmed that lower CVP can result in increased CO, which may improve 28-day mortality in specific patients with circulatory shock. Higher CO caused by a reduction in CVP may also contribute to renal function improvement. We found that, in some patients, higher CO derived from the reduction in CVP can benefit from EVLWI decreases and renal function improvement through inotrope and dehydration treatment. Choosing fluid therapy for these patients requires careful choice.

It has been acknowledged that CVP should not be used as the hemodynamic response to a fluid challenge (18, 19). However, increasing numbers of studies recognize that CVP may be an indicator of poor outcomes. In the VASST study, Boyd et al. (7) found that fluid overload and increased CVP (>12 mmHg) caused an increase in mortality in critically ill patients. Danziger et al. found that peripheral edema affects the prognosis of critically ill patients. Moreover, CVP >13 mmHg increased the adjusted risk of hospitalization up to 35% compared with CVP <7 mmHg (20). In addition, CVP increases of 1 mmHg may increase the risk of hospitalization by 2% (21). Legrand et al. (5) showed a linear relationship between the risk of acute kidney injury (AKI) and CVP in a retrospective sepsis cohort. In our study, we demonstrated an association between lower CVP and lower 28-day mortality (Figure 2A). To reveal the effects of the CVP and CO relationship on prognosis, the patients were divided into four groups. We showed that the CVP↓+ CO↑ group had a higher 28-day survival rate (Figure 2B). Renal function (SCr and BUN) in the CVP↓+ CO↑ group was significantly better 72 h after PICCO (Figure 4). One potential mechanism of lower CVP and higher survival rate may be that reduced pressure of the VR can improve kidney congestion (22). Another possible mechanism may be that elevated CVP may influence pulmonary circulation and oxygenation, and MV, in turn, may affect CVP (6, 23, 24). In addition, there may be an effect of CVP on microcirculation perfusion (25), cerebral blood flow regulation (26), and other organ/tissue perfusion. Therefore, appropriately lower CVP levels are conducive to maintaining normal physiological organ function.

**Figure 5:**
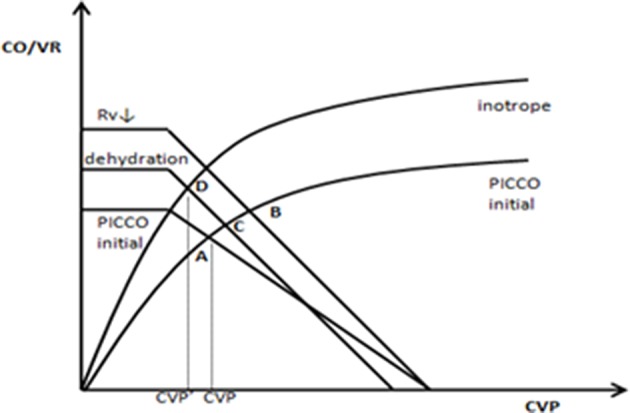
A possible hemodynamic mechanism for higher CO induced by CVP reduction.

Based on the abovementioned factors, lower CVP is better. However, in the pathophysiological state, we must obtain a suitable CO matched with the lower CVP. Therefore, a heart function curve must be used to illustrate the hemodynamic characteristics of higher CO induced by CVP reduction. In this study, we found that the CVP↓+ CO↑ group had lower CFI and higher EVLWI at PICCO initiation. Twenty-four hours after PICCO, the CFI significantly increased, and EVLWI decreased. Inotrope use and dehydration perhaps produced hemodynamic effects based on the Starling-Guyton theory (27, 28). As shown in Figure 5, cardiotonic drugs, including the vasodilators dobutamine and milrinone, may cause decreased venous resistance (Rv), resulting in increases in the slope of the VR curve (A → B). Additionally, dehydration reduced the stress volume and lowered the MSFP (B → C). The positive inotropic effect further caused the Starling curve to increase (C → D). The final integrated effect was the induction of higher CO by CVP reduction. From the comparison results of the intervention process (Table 2), cardiotonic drug use in the CVP↓+ CO↑ group was greater than that in the remaining three groups. Moreover, the CVP↓+ CO↑ group had a negative fluid balance.

Recently, accumulating evidence has suggested that persistent positive fluid balance is associated with higher mortality in sepsis (29, 30). However, few studies have directly demonstrated the benefits of dehydration in ARDS patients (31). Are there critically ill patients who can benefit from dehydration? Based on perfusion index satisfaction, the answer is yes. In this study, the CVP↓+ CO↑ group achieved a negative fluid balance. Although the CVP↓+ CO↓ group also had a negative fluid balance, its lactate levels increased after dehydration. Generally, it is expected that hypoperfusion does not occur when the plasma refilling rate is adequate for hypovolemia prevention. Excessive fluid removal must be avoided. Our results proved that a negative fluid balance could be achieved during circulation stabilization in some patients with adequate perfusion assisted by vasopressive and inotropic drugs. We proposed a hypothesis that negative fluid resuscitation may be useful and necessary in some specific situations of shock and in the later stages of fluid resuscitation, which we defined as “negative fluid resuscitation (NFR)” (32).

This study has some limitations. First, it was a single-center, retrospective cohort study including only patients under PICCO monitoring and treatment. In addition, the sample size is limited. More importantly, we cannot exclude the differences in the indications of different patients when PICCO is placed. The selection bias of why, when, and how to use PICCO catheterization for severely ill patients may affect the final conclusion (33). Second, septic shock patients often have cardiac dysfunction; therefore, the use of vasoactive drugs is common. Treatment with cardiotonic medications can give only qualitative, not quantitative, results. However, the effect of vasoactive drugs on CVP and CO is not clear. Finally, the effect of blood flow, pressure, and drug use on venous tension was not fully described due to technical limitations. We cannot directly measure the resistance of the venous system. Therefore, in summary, prospective randomized control studies should be conducted in the future if possible.

In general, this study challenges high-volume resuscitation, which is frequently used in everyday practice. We found that lower CVP is associated with a good prognosis, especially in patients with higher CO derived from CVP reduction, because these patients can exhibit EVLWI-level reduction, cardiac dysfunction amelioration, and renal function improvement through inotrope and dehydration treatment. The findings of this study can broaden the knowledge of such patients in clinical practice, allowing us to reduce CVP and acquire benefits.

## Data Availability Statement

The data used to support the findings of this study were provided by DLiu and are under license; thus, they cannot be made freely available. Access to these data will be considered by the author upon request, with permission from the Department of Critical Care Medicine, Peking Union Medical College Hospital.

## Ethics Statement

The studies involving human participants were reviewed and approved by Ethics Committee of Peking Union Medical College Hospital. The patients/participants provided their written informed consent to participate in this study. Written informed consent was obtained from the individual(s) for the publication of any potentially identifiable images or data included in this article.

## Author Contributions

DLiu and LS: conception and design. DLi and QZ: data extraction. PP and XZ: statistical analysis. LS and PP: interpretation of data and writing. DLiu, XW, and YL: review and revision of the manuscript.

### Conflict of Interest

The authors declare that the research was conducted in the absence of any commercial or financial relationships that could be construed as a potential conflict of interest.

## Abbreviations

CVP: central venous pressure
ARDS: acute respiratory distress syndrome
VR: venous return
CO: cardiac output
MAP: mean arterial pressure
CI: cardiac output index
CFI: cardiac function index
EVLWI: extravascular lung water index
SD: standard deviations
AKI: acute kidney injury.
